# Impact of HIV-related stigma on treatment adherence: systematic review and meta-synthesis

**DOI:** 10.7448/IAS.16.3.18640

**Published:** 2013-11-13

**Authors:** Ingrid T Katz, Annemarie E Ryu, Afiachukwu G Onuegbu, Christina Psaros, Sheri D Weiser, David R Bangsberg, Alexander C Tsai

**Affiliations:** 1 Connors Center for Women's Health and Gender Biology, Brigham and Women's Hospital, Boston, MA, United States; 2 Center for Global Health, Massachusetts General Hospital, Boston, MA, United States; 3 Harvard Medical School, Boston, MA, United States; 4 Harvard College, Cambridge, MA, United States; 5 Harvard School of Public Health, Boston, MA, United States; 6 Department of Psychiatry, Massachusetts General Hospital, Boston, MA, United States; 7 Division of HIV/AIDS, San Francisco General Hospital, University of California at San Francisco, California, United States; 8 Mbarara University of Science and Technology, Mbarara, Uganda

**Keywords:** HIV, stigma, disclosure, adherence, social support, poverty

## Abstract

**Introduction:**

Adherence to HIV antiretroviral therapy (ART) is a critical determinant of HIV-1 RNA viral suppression and health outcomes. It is generally accepted that HIV-related stigma is correlated with factors that may undermine ART adherence, but its relationship with ART adherence itself is not well established. We therefore undertook this review to systematically assess the relationship between HIV-related stigma and ART adherence.

**Methods:**

We searched nine electronic databases for published and unpublished literature, with no language restrictions. First we screened the titles and abstracts for studies that potentially contained data on ART adherence. Then we reviewed the full text of these studies to identify articles that reported data on the relationship between ART adherence and either HIV-related stigma or serostatus disclosure. We used the method of meta-synthesis to summarize the findings from the qualitative studies.

**Results:**

Our search protocol yielded 14,854 initial records. After eliminating duplicates and screening the titles and abstracts, we retrieved the full text of 960 journal articles, dissertations and unpublished conference abstracts for review. We included 75 studies conducted among 26,715 HIV-positive persons living in 32 countries worldwide, with less representation of work from Eastern Europe and Central Asia. Among the 34 qualitative studies, our meta-synthesis identified five distinct third-order labels through an inductive process that we categorized as themes and organized in a conceptual model spanning intrapersonal, interpersonal and structural levels. HIV-related stigma undermined ART adherence by compromising general psychological processes, such as adaptive coping and social support. We also identified psychological processes specific to HIV-positive persons driven by predominant stigmatizing attitudes and which undermined adherence, such as internalized stigma and concealment. Adaptive coping and social support were critical determinants of participants’ ability to overcome the structural and economic barriers associated with poverty in order to successfully adhere to ART. Among the 41 quantitative studies, 24 of 33 cross-sectional studies (71%) reported a positive finding between HIV stigma and ART non-adherence, while 6 of 7 longitudinal studies (86%) reported a null finding (Pearson's *χ* ^2^=7.7; *p*=0.005).

**Conclusions:**

We found that HIV-related stigma compromised participants’ abilities to successfully adhere to ART. Interventions to reduce stigma should target multiple levels of influence (intrapersonal, interpersonal and structural) in order to have maximum effectiveness on improving ART adherence.

## Introduction

Adherence to HIV antiretroviral therapy (ART) is a critical determinant of HIV-1 RNA viral suppression and health outcomes [1–3]. Early studies of ART adherence focused primarily on cognitive processes that may affect adherence, such as forgetfulness and health literacy [4–6]. More recently, investigators have shown that ART adherence in resource-limited settings, where treatment is generally provided free of charge, may be contingent upon structural barriers, such as food insecurity [7–12] or geographic isolation and lack of resources to pay for transportation to clinic [13–17].

The stigma of HIV and AIDS is one social process that has been broadly assumed to adversely affect multiple facets of engagement in HIV-related care as well as other factors that may undermine ART adherence, including HIV serostatus disclosure [18–20], social support [18, 21] and mental wellbeing [21, 22]. Goffman [23] conceptualized stigma as an “attribute that is deeply discrediting” imposed by society that reduces someone “from a whole and usual person to a tainted, discounted one” (p. 3). When the attribute becomes linked to “discrediting dispositions” (e.g., negative evaluations or stereotypes), these may come to be widely believed in the community [24]. During the labelling process [25–27], persons with and without the stigmatized attribute are separated into “them” and “us” [28] and may be subjected to overt acts of hostility and discrimination (enacted stigma) [29]. To avoid the potentially unpleasant consequences of revealing their discredited status, stigmatized persons may elect to conceal their seropositivity from others [20, 30]. Stigmatized persons may also internalize the beliefs held in the community and develop self-defacing internal representations of themselves (internalized stigma) – possibly leading to demoralization, diminished self-efficacy and emotional distress [31, 32].

Despite substantive advances in our understanding of the stigma process, the mechanisms through which stigma compromises ART adherence are not well understood. From a public health perspective, this is an important gap in the literature because sustained adherence [33] is a critical step in the spectrum of engagement in HIV-related care [34, 35]. Although the “test-and-treat” approach [36] has achieved a great deal of popularity in a brief amount of time, observers have expressed concerns that persisting stigma may pose a major obstacle to its success [37]. Therefore, we undertook this review to systematically assess the relationship between HIV-related stigma and ART adherence.

## Methods

### Search strategy and study selection

Three study authors (AER, AGO, ACT) searched nine electronic databases for published and unpublished literature: BIOSIS Previews, the Cumulative Index to Nursing and Allied Health Literature (CINAHL), Embase, the Educational Resources Information Center (ERIC), the Medical Literature Analysis and Retrieval System Online (MEDLINE), ProQuest Dissertations & Theses, PsycINFO, Web of Science (Science Citation Index Expanded, Social Sciences Citation Index, and Arts & Humanities Citation Index) and the World Health Organization African Index Medicus. In general, each set of search terms applied to these databases was oriented towards identifying studies of ART adherence among HIV-positive adults (Box S1). We conducted all searches in May 2011, with the exception of the ProQuest search, which was performed in June 2011. In February 2013, one study author (ACT) updated the MEDLINE search to identify more recent articles published since the study was initiated. We also consulted with experts in the field to identify additional studies that our systematic evidence search may have missed.

First we imported all records into EndNote reference management software (version X4.0.2, Thomson Reuters, Philadelphia, Penn.) and used the automated “Find Duplicates” function to exclude any duplicates. Then we screened the titles and abstracts of all records to identify studies that appeared to be potentially related to ART adherence among HIV-positive persons. We then obtained the full text of these articles for review, specifically to identify articles that reported either a quantitative estimate of association between a measure of stigma or disclosure and a measure of adherence, or qualitative findings about how stigma or lack of disclosure affected adherence. Although our review was focused on the relationship between stigma and adherence, we also chose to include studies examining the impacts of serostatus non-disclosure because it is a proximate consequence of stigma [19, 20]. Our goal in including qualitative studies as part of this systematic review was to inductively develop an in-depth understanding of persistent themes and assess the transferability of these themes across contexts [38]. Due to our interest in describing relationships between stigma and adherence across a wide range of countries, we chose not to exclude any study based on quality, country of origin or language.

### Quality assessment

To assess the quality of the included qualitative studies, we adapted questions representing the three key conceptual domains described in the Critical Appraisal Skills Programme quality assessment tool [39, 40]. These domains also mapped onto prominent criteria employed by previous researchers as identified in the review of qualitative quality assessment tools by Tong *et al*. [41]. The criteria we used were as follows: (1) the role of the researcher was clearly described; (2) the sampling method was clearly described; (3) the method of data collection was clearly described; and (4) the method of analysis was clearly described. We found that the included qualitative studies consistently described the role of the research and the method of data collection, but many studies reported neither the sampling method nor the method of analysis. Overall, 15 studies were assessed to be at low risk of bias (Table S1).

To assess the quality of the included quantitative studies, we developed an assessment tool based on the six major conceptual domains identified by Sanderson *et al*. [42]. The criteria we used were as follows: (1) the study was based on a probability sample of participants; (2) the study used a validated self-report scale to measure stigma or disclosure; (3) the study used a validated self-report scale or objective count (e.g., pill count, pharmacy refill) to measure ART adherence; (4) the statistical analysis accounts for missingness at random (MAR) or missingness not at random (MNAR) (longitudinal studies only); (5) the study design or statistical analysis controls or adjusts for potential confounding; and (6) competing interests were declared. Overall, all studies except for one were assessed to be at risk of bias (Table S2).

### Data synthesis

We organized studies by year of publication, country of origin, study design and types of measures employed. For the quantitative studies, due to substantial heterogeneity in the measures of stigma, serostatus disclosure and ART adherence that were employed, we did not attempt to summarize the data using meta-analysis. However, we examined patterns across studies with respect to the estimated associations and the precision of these estimates.

For the subset of qualitative studies, our goal was to generate new theoretical insights. Therefore, we used the iterative process of meta-synthesis proposed by Noblit and Hare [43] to identify themes that recurred frequently or were prominently featured throughout the data. Meta-synthesis (also described as meta-ethnography) is an interpretive approach to summarizing qualitative research that has been employed to understand vaginal practices in sub-Saharan Africa [44], delays in presentation for cancer care [45] and adherence to tuberculosis treatment [46]. Key themes and concepts were collected and peer-reviewed for inclusiveness. First-order findings (quotations) were used to support second-order interpretations (authors’ analyses) to gain new insight into the relationships between stigma and ART adherence. A summary definition of second-order constructs was generated for further clarification and then consolidated into a line of argument that led to a third-order analysis, which we describe below. Based upon the data set, we achieved theoretical saturation within the first 10 manuscripts, although basic elements for meta-themes were evident as early as six manuscripts. Variability within the data followed similar patterns, consistent with prior qualitative meta-synthesis research [47].

## Results

**Figure 1:**
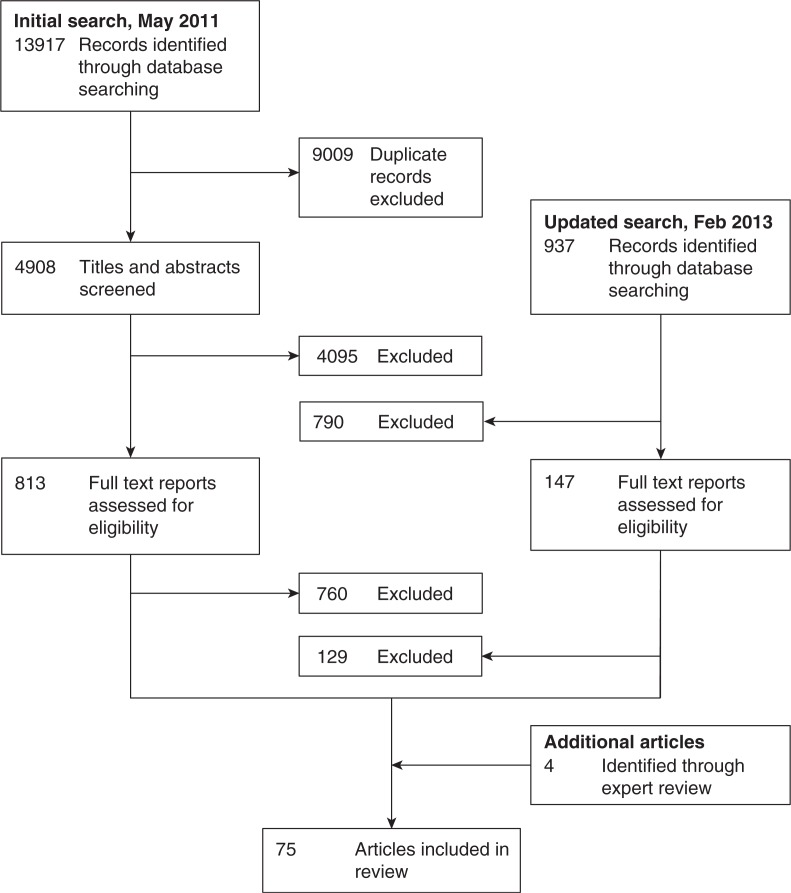
Flow diagram. We identified 14,854 records by searching nine electronic databases, yielding 34 qualitative studies and 41 quantitative studies.

Our initial search yielded 14,854 records, of which 9009 were identified as duplicates through the use of automated software (Figure 1). After screening the titles and abstracts of the remaining 5845 records, we eliminated 4000 records that did not appear to contain relevant data on adherence or provided potentially relevant adherence data specific to a specialized population (e.g., children or pregnant women), eight unpublished conference abstracts or dissertations matched to subsequently published peer-reviewed journal articles in our database of records, 199 reviews that did not report original data, and 678 additional duplicates that had been misclassified as non-duplicates by the automated software. We retrieved 960 journal articles, unpublished dissertations and conference abstracts for full text review. Of these, 889 did not contain quantitative or qualitative data relating stigma or disclosure to ART adherence and were therefore excluded. Expert review suggested four additional articles for inclusion. The final sample included 75 studies: 34 qualitative studies and 41 quantitative studies.

### Synthesis of qualitative studies

Thirty-four qualitative studies conducted during 1999–2013 were included in the review, including one written in French. Represented in these manuscripts were views from 1328 study participants in 26 countries. Of note, only one country from the UNAIDS Eastern Europe and Central Asia region was represented: Serbia and Montenegro. The median number of participants was 38 (interquartile range (IQR), 27 to 48; range, 6 to 118). Participants included adult men and women ranging in age from 18 years to over 60 years old, HIV-positive persons as well as providers of HIV care, single persons and those in intimate partnerships, and persons with and without children. Specific high-risk groups were well represented and included men who have sex with men, injection drug users and commercial sex workers.

After reviewing each of the qualitative studies in detail, we identified 24 second-order constructs, supported by original quotes, in multiple manuscripts. Second-order constructs relevant to ART adherence were identified, and key themes were generated into a line of argument that led to 15 third-order constructs. These were grouped into five distinct third-order labels that we categorized as themes, all of which are described in detail in Table 1.

**Table 1:** Qualitative studies on stigma, disclosure and ART adherence (*N*=34)

Third-order labels	Third-order constructs	Second-order constructs	Summary definition	First-order constructs	Source(s)
Social support	Intimate and familial relationships	Spousal, peer and familial support	Participants discussed support from spouses, peers and family as critical for overcoming stigma and maintaining adherence, as was having a sense of obligation to family	*Well, they encourage me, like my folks have [said] ‘you took your medication today?’* [55, p. 5] *I am thankful to God for giving me such a good husband. He takes care of me well. I have given him a lot of trouble. He has spent so much money for my treatment*. [54, p. 496]	[48–70, 78, 79]
		Context of male-dominated household decision-making	In cultures where men are typically heads of their households, women fear disclosing their serostatus as they fear social isolation and abandonment. Women may choose to have providers give the test information to their husbands by bringing them in for testing. In addition, in some cultures, women cannot travel alone to clinic to pick up their medications.	*[After testing positive] I went back home and first kept quiet for two days. I asked myself, how can I approach him to tell him? One day when he came back, I told him, they checked my blood but they refused to give me the results until I take my spouse in for testing. I convinced him and he accompanied me*. [57, p. S88]	[49, 52, 54, 57, 64, 68, 72]
		Healthy children reducing stigma	Clinical response to ART in children of HIV-positive mothers reduces stigma and often re-establishes mother's role in family	*Then when she saw that since giving birth, my baby was not falling sick (the other children used to be sickly), that my baby was looking nice, did not have a rash, and was growing fast she said ‘I used to think you were infected. I had taken you out of all of my plans.’* *I responded that, ‘I am not infected, don't you see my baby?’ So that's where I ended her suspicions about my being sick. Now she knows that I am not infected, which is not true*. [57, p. S88]	[57]
	Compromised relationships	Physical manifestations of HIV and AIDS leads to social isolation	Physical signs of ill health may lead to abandonment or to the belief that the HIV-positive person is already dead	*These days when people come to know that you have AIDS they don't want to come near you, as if you are an abominable thing (‘bakwenyinyala’). You cannot feel free. Wherever you go they start talking, ‘See that one, she is sick.’* [57, p. S88])	[55–57, 64–67, 69, 71, 72]
		Complex regimens with large numbers of medications	Complex regimens characterized by a large pill burden that required undesired disclosure in order to adhere	*… things got messed up, like my schedule, wherever you go, you got to bring the medicine pack, it's even upsetting to open a bunch of medicines*. [53, p. 3] *Our guests were at my home; I didn't feel comfortable pulling out my drug boxes, then I forgot and missed my drugs*. [74, p. 467]	[12, 52, 53, 55, 59–61, 63–65, 69, 72–74]
		Social rejection	Participants adopted strategies of concealment because they feared ridicule or discrimination if they disclosed their HIV status or if they were seen taking their medications	*My company made it hard. You know, because I felt like I had to hide my medicine, you know? All, you know, for shame*. [55, p. 5] *Ordinary public thinks that if they mingle along with the patient means they will get HIV*. [48, p. 532]	[12, 48, 49, 54–56, 59, 64–72, 76, 77]
		Treatment side-effects	Observable side-effects of medications (e.g., dysmorphic body changes) carried stigma	*It wasn't hard for me to take my medicines; it was the things that people would say …* [55, p. 5] *The medications compounded the way I felt, how badly I felt, but I kept taking them because I knew it was temporary*. [74, p. 466]	[12, 53, 55, 56, 60, 61, 63–66, 68, 71, 73, 74, 76]
	Negotiating disclosure to a child	Stigma associated with a child's HIV status	Maternal shame and stigma related to perinatal acquisition of HIV kept them from informing HIV-positive children about their seropositivity, with attendant challenges in ART adherence	*The thing that disturbs me is that I always think what will I tell my child when he grows to a level of understanding and he asks me why he is taking drugs. Because even now he asks me, ‘Mummy I no longer cough but why am I still taking drugs every day?’ What will I tell the child?’* [57, p. S88]	[48, 53, 57, 64]
Self-Identity	Race/minority status	Outsider status based on race	HIV-positive persons who belonged to racial minority groups felt further stigmatized and socially isolated		[49, 55]
	Sexual orientation/relationship status	Impact of social norms on stigma and willingness to disclose	Social norms further stigmatized HIV-positive persons if the mode of acquisition was not regarded as socially acceptable behavior	*In the gay community, I can't go to somebody and say, ‘I'm HIV.’ People avoid the subject. They do not disclose it*. [51, p. 906]	[50, 51, 54, 61–63, 71–74, 76, 77]
	Substance abuse	Social marginalization of injection drug use intensified for HIV-positive users	Participants who actively used illicit substances discussed being unable to establish relationships with HIV-negative persons or non-injection drug users, and feeling socially isolated	*Drug users, it's a group that right now everyone in society hates. Including myself, I hate myself. But the problem is [that] there is nothing I can do*. [77, p. 1244]	[51, 77]
	Redefining healthy living	Self-perception as pro-active/choosing to be healthy	Participants described knowing friends who died from AIDS and not wanting to be like them; the notion of “choosing to live” [74, p. 466]	*Then I had some friends die of full-blown AIDS, and I looked around and seen what a horrible death that was* … *And so I know I wanted to live, and I wouldn't want to send my family through that. So I knew I had to take my medicine*. [55, p .4] *I didn't want to start drugs, but I had seen two AIDS patients dead. They hadn't used drugs*. [74, p. 466]	[52–56, 58, 59, 61, 66, 70, 72–74]
	Acceptance of status	Self-identifying as someone who is HIV-positive	Participants who had accepted their status found it easier to adhere vs. those who had difficulty taking medications because it reminded them of their seropositivity	*The thing is it's my life, you know. I don't see it much if somebody comes to me and tells me that, ‘you've got HIV – you are HIV’. I don't have a problem with that because that's not his problem, that's my problem you know. As long as I know how I manage it, I don't give a damn about any other person*. [56, p. 303]	[50, 56, 67, 69, 70, 73, 74]
Poverty	Economic implications of HIV	Mutually reinforcing relationship between poverty and stigma	HIV-related illness and perceived economic inadequacy leading to social exclusion	*They see it as useless to assist someone who has a shorter time to live. It's like wasting money. Why assist someone who is going to die?* [67, p. 1311] *There is no need to waste any more money on her, give me this lady and I will put her in the car and take her to her rural home with her children*. [72, p. 875] *With ART, I have returned to work and earn money; friends who avoided me in the past are now more accepting of me* … *If I do not take this medicine as I am told, I will get sick and*	[54, 56, 67, 72]
				*won't be able to work again. People will also begin to avoid me again*. [72, p. 877]	
			Economic insecurity resulting from HIV-related stigma	“I thought that people would know my HIV status when I have illnesses regularly and am out of the office several times.” [67, p. 1311]	[54, 67, 72]
		Costs associated with treatment	Costs associated with purchasing medications or with travel to the treatment centre (along with loss of wages) made even free ART prohibitively expensive for some, leading to treatment interruptions	*Even if I go for work I get Rs 100 in which 60 goes for tablets. So in the rest I have to manage the other expenses, which is very difficult. Medicines for HIV infection should be like other general medicines where everyone can afford to buy. Now I am not sure I can continue the treatment for a long time*. [48, p. 529]	[12, 48, 54, 60, 61, 64, 67, 68, 70, 72, 76, 77]
Coping	Maladaptive strategies	Anger at diagnosis	Inability to accept diagnosis and anger at diagnosis, with associated inability to engage in HIV care and adhere to ART	*I was mad, and I was upset, and I was in denial. And it took me five years to tell anybody that was close to me. So I kept that to myself for a long time, and I was very angry. Right now, I still don't take [the medicines] like I should*. [55, p. 4]	[55, 72]
		Substance use and abuse	Consumption of alcohol and use of drugs provided a temporary refuge but also made ART adherence more difficult	… *I began to skip the medication. I said to myself, ‘Well, today I'm not taking it, ‘cause I'm gonna party* … *[drink] Come on, I was born to party* … [53, p. 3]	[52, 53, 59, 73, 78]
		Fear that drugs are dangerous and/or that HIV is a curse fuelled by stigma	Participants expressed concerns about taking medications feared to be dangerous or toxic	*Rural people do still not believe this medicine [ART] works for HIV patients. HIV people will die eventually either taking or not taking ART. Why should I die by taking these malicious pills?* [68, p. 3]	[12, 68, 71, 72]
	Acceptance	Knowledge that taking medications will provide benefits	Acceptance of the diagnosis counter-balanced stigma, as participants described moving on a continuum from willingness to take medications, to engagement in pro-active healthy lifestyle changes	*This is your own responsibility. You know what you got. You know you got medicine to take. No matter what nobody else say or how peoples feel about it, you got to take care of yourself first*. [55, p. 4]	[54–56, 58, 59, 66, 67, 69, 70, 72–74]
				*During [the] last 5 years, taking medications showed me its benefits. My CD4 cells [sic] count was 80, with high viral loads, but now I am okay. They actually helped and gave me more longevity*. [74, p. 467]	
	Mental wellbeing	Treatment of depression and anxiety related to diagnosis	Treatment of depression resulting from HIV diagnosis could ameliorate stigma and social isolation		[49, 57, 65, 67, 69, 72, 73, 77]
	Morality and spirituality	Notion of God's will	Participants discussed relinquishing control of their lives to God and putting their faith in a higher power to help them overcome adversity	*I just want to be a living witness, that God has all power. He can do all things, and I put my faith and trust in Him*. [55, p. 4–5] *I believe in the power of prayers – I believe in my church. It's got hope for me … because I have a feeling that God loves us … God is the person that gave you that disease, and God is the person who can take it out from you* … *You have to have faith in that*. [56, p. 305]	[12, 52, 54–61, 67, 69, 72]
Health systems	Importance placed in clinical support staff	Nursing and physician support to gain trust and overcome social isolation associated with stigma	Programs supporting social support and building trust with the adherence nurse or doctor were described as essential for people who reported stigma as a barrier to ART adherence	*I felt so alone. It's nice to know that somebody does understand what it is all about and you can depend on that person*. [75, p. 117] *I trust the doctors and nurses. Therefore I started the drugs*. [74, p. 466]	[50, 55, 58–60, 62, 63, 67, 69, 70, 72–75, 80]
		Support in designing tolerable combination of medications that are easily available	Participants felt it was easiest to adhere if they were on tolerable medications and if providers were available in the event of adverse side effects vs. those who feared taking medications because of potential side effects or complications. It was also important to ensure that there were no stock-outs and that medications were easily available.	*I didn't know the advantages of medications, I feared the complications; therefore, I started it very late. Actually, it was [a] wasting of my time*. [74, p. 466] *We can't have any plan, because we don't know when supplies will fail. Some people can get medicine and some can't*. [80, p. 317]	[55, 58–60, 73, 74, 80]
	Family-driven treatment	Establishing treatment for all members of the household	Treatment to all HIV-positive members of a family (including spouse and children) provided support to overcome stigma and improve medication adherence		[54, 57]

#### Theme 1: social support

The most commonly cited theme related to ART adherence was the role of social support. Specifically, participants described spousal or familial support as being critical for enabling them to overcome enactments of HIV-related stigma and other obstacles to care and successfully adhere to treatment [48–70]. As noted by one 45 year-old HIV-positive rice dealer in Chennai, India,

A person without a family is like a single tree struggling for life. My children and my wife are my backbone. Now I have brought changes in myself and want to achieve many things. [54, p. 496]

Compromised relationships could result from either HIV illness or HIV treatment. Many participants described being socially isolated due to the physical manifestations of HIV-related illness [55–57, 64–67, 69, 71, 72] . As described by one HIV-positive mother in Kampala, Uganda,

These days when people come to know that you have AIDS they don't want to come near you, as if you are an abominable thing (‘bakwenyinyala’). You cannot feel free. Wherever you go they start talking, ‘See that one, she is sick’. [57, p. S88]

On the other hand, HIV treatment could also undermine social relationships. Unintended disclosure was viewed as a consequence of being on complex regimens that often needed to be taken multiple times per day [12, 52, 53, 55, 59–61, 63–65, 69, 72–74]. This was commonly discussed in some of the older studies, which were conducted during a time when pill burden was high and participants reported difficulty in understanding when and how to take their medications [12, 50, 52, 58, 60, 61, 64, 67, 68, 70, 74, 75]. Attempts at concealment, such as by hiding medications or furtively taking medications, were described as contributing to treatment interruptions [12, 48, 49, 54–56, 64–72, 76, 77].

In addition, some participants felt that the medications themselves were associated with side effects that had unwelcome physical manifestations:

[ART] has given more side-effects for me such as vomiting, herpes/zoster, and skin rashes. I have lost my sight in my right eye and my left eye also has poor vision.

– HIV-positive woman from far western Nepal [68, p. 7]

Desire to avoid these physical stigmas, or fear of “the thing [sic] that people would say” [55, p. 102], motivated some participants to avoid taking medications and evade detection.

A more circumscribed discussion in the literature related to norms about gender roles, particularly in patriarchal cultures. Byakika-Tusiime *et al*. [57] explained how HIV-positive women were better able to adhere to ART when others did not identify them as being infected with HIV. An HIV-positive mother could evade detection by giving birth to an uninfected child and establishing her role as a caretaker. This was discussed by an HIV-positive mother in Kampala, Uganda, who described how giving birth to a healthy baby changed her family's assumptions about the inevitability of her death:

When [my sister] saw that since giving birth, my baby was not falling sick (the other children used to be sickly), that my baby was looking nice, did not have a rash, and was growing fast she said ‘I used to think you were infected. I had taken you out of all my plans.’ I responded that ‘I am not infected, don't you see my baby?’ So that's where I ended her suspicions about my being sick. Now she knows that I am not infected, which is not true. [57, p. S88]

Other authors mentioned the importance of women being able to hide their seropositivity in settings where men dominated household decision-making, so as to avoid social isolation and/or abandonment [49, 52, 54, 64, 68, 72]. In these settings, some women reported relying on healthcare providers to inform their sexual partners of their HIV status rather than informing their partners directly themselves.

Women who gave birth to an HIV-positive child experienced feelings of shame and social rejection, both within and outside of the family. Participants in these studies discussed the difficulty associated with disclosing the status of an HIV-positive child, particularly in communities where HIV was highly stigmatized and where appearing ill often led to abandonment by one's family and community [48, 53, 55–57, 64–67, 69, 71, 72] .

The thing that disturbs me is that I always think what will I tell my child when he grows to a level of understanding and he asks me why he is taking drugs. Because even now he asks me, ‘Mummy, I no longer cough but why am I still taking drugs every day?’ What will I tell the child?’

– HIV-positive mother from Kampala, Uganda [57, p. S88]

#### Theme 2: self-identity

Self-identity was another prominent theme identified in these studies. Multiple studies elaborated on how social norms intensified the stigma of HIV and undercut participants’ willingness to disclose to others [50, 51, 54, 61–63, 71–74, 76, 77] . In many settings, study participants described HIV-related stigma as being layered on top of pre-existing inequalities, such as those related to gender, race or sexual minority status:

I often hear my friends speak negatively about people being HIV-positive. They always have degrading or negative remarks to make. What I dislike most is when they call people names (e.g., fagot, whore, and junkie). Whenever I go out with them or they come over to visit, I don't take my medications. I could never let them know I'm positive.

– HIV-positive African-American woman living in Baltimore, U.S. [49, p. 684]

Konkle-Parker *et al*. [55] and Edwards [49] both discussed the difficulty that persons in a minority group experienced when self-identifying as HIV-positive, since it often led to further enactments of stigma, including overt discrimination and/or acts of hostility. In such a setting (and consistent with Theme 1), many participants opted not to take their medications for fear of disclosure. Ware *et al*. [51] and Sabin *et al*. [77] described the added burden and social isolation that accompanied an HIV diagnosis among participants who actively used illicit substances. In these cases, self-efficacy was often low, and the lifestyle modifications required to achieve consistent adherence proved to be challenging for participants.

Drug users, it's a group that right now everyone in society hates. Including myself, I hate myself. But the problem is [that] there is nothing I can do.

– 40-year-old, injection drug using, HIV-positive married man living in Old Dali, Yunnan Province, China [77, p. 1244]

The experiences of persons who had internalized the stigma of HIV was contrasted with reports of persons who had accepted their HIV status and who had successfully cultivated a self-perception of being pro-active and “choosing to live” [74, p. 466]. These participants were able to successfully adhere to their ART regimens [52–56, 58, 59, 61, 66, 72–74] . In these studies, participants described how the deaths of HIV-positive friends motivated them to take responsibility for their own treatment. Some participants also described feeling strong enough to continue to work and provide for their families.

Then I had some friends die of full-blown AIDS, and I looked around and seen what a horrible death that was … And so I know I wanted to live, and I wouldn't want to send my family through that. So I knew I had to take my medicine and … I know I wants to live

– HIV-positive African-American study participant from Mississippi [55, p. 4]

#### Theme 3: poverty

**Figure 2:**
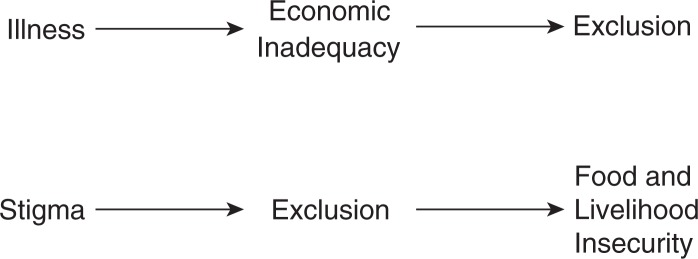
Reciprocal relationships between poverty and stigma. HIV-associated illness reinforces the perceived economic inadequacy of HIV-positive persons, who are excluded from networks of mutual aid. Stigmatized persons are excluded from the community, undermining their social support and worsening economic insecurity.

In several studies, participants also described how poverty and stigma were intertwined in a reciprocal and mutually reinforcing relationship (Figure 2). Participants spoke of being viewed as weak, unproductive members of society and of being excluded from informal networks of mutual aid:

They see it as useless to assist someone who has a shorter time to live. It's like wasting money. Why assist someone who is going to die?

– HIV-positive person living in Dar es Salaam, Tanzania [67, p. 1311]

Thus, conditions of poverty worsened stigma by emphasizing one's economic worth (or lack thereof) to the community. In resource-limited settings where social networks serve as a form of informal risk-sharing (consistent with Theme 1), and where neighbours often live in close proximity to each other, participants reported feeling ashamed and ultimately more stigmatized by the public nature of unwanted disclosures:

I used to have a neighbour … who knew my status. At times, I used to get porridge from KENWA and bring it home. She had a child who was my kid's friend and age mate. One day, I gave the porridge to her child and [she] was furious and shouted at the little girl; ‘where did you get that porridge? Take it back! You are taking porridge from people with AIDS,’ she was shouting outside and I was in the house.

– HIV-positive woman living in a slum community in Nairobi, Kenya [72, p. 874]

Conversely, stigma was also found to exacerbate the economic impacts of HIV. Economic insecurity resulting from stigma and social isolation was particularly challenging for widowed women who had lost their husbands to AIDS. Tarakeshwar *et al*. [54] described 9 out of 10 widowed women living in Chennai, India, who were discriminated against, experienced housing insecurity and were isolated by their in-laws after their husbands’ deaths. Stigma was also cited as leading to embarrassment at work, and ultimately causing participants to stop working in order to avoid disclosure, leading to further economic insecurity:

I was on 5 days leave [when I came to test for HIV] and I stayed another week. They were looking for me at work … I was staying [away] because I was sort of embarrassed by my own things. I was embarrassed by my own fate.

– 39-year-old HIV-positive unmarried man living in Gaborone, Botswana [56, p. 304]

Lastly, for participants in resource-limited settings, financial burdens posed a significant barrier to adherence due to costs of the medications themselves, the costs of transportation to pick up free medications from clinic, or wages foregone when attending clinic [12, 48, 54, 60, 61, 64, 67, 68, 70, 72, 76, 77]. These treatment interruptions further compromised participants’ health, reinforcing their status as unproductive members of the community.

#### Theme 4: coping

Coping emerged as a means by which participants attempted to manage stigma and adhere to ART. At times, these coping strategies were maladaptive and detrimental to health. Many participants reported low self-esteem, depressed mood or anger related to their diagnosis, citing their inability to cope with their HIV status as the reason they failed to take their medications [49, 55, 57, 65, 67, 69, 72, 73, 77]:

I was mad, and I was upset, and I was in denial. And it took me five years to tell anybody that was close to me. So I kept that to myself for a long time, and I was very angry. Right now, I still don't take [the medicines] like I should.

– HIV-positive study participant recruited from a large public infectious disease clinic in Mississippi [55, p. 4]

In addition, ART misconceptions (e.g., “Why should I die by taking these malicious pills?”[68, p. 3]) and HIV conspiracy beliefs that were often fuelled by stigma led to ART non-adherence [12, 68, 71, 72]. Participants who lacked the internal resources to cope adaptively described how they self-medicated with alcohol or illicit substances, but these behaviours further compromised their abilities to consistently adhere to treatment [52, 53, 73].

Adaptive coping strategies included those that supported adequate treatment for depression and anxiety, along with acceptance of one's diagnosis. These strategies appeared to provide a protective buffer against stigma and promote acceptance of lifelong treatment [12, 54–56, 58, 61, 67, 69, 72–74] , particularly for those who were able to incorporate these into their new self-identities (consistent with Theme 2). Likewise, spirituality and faith in God enabled some participants to overcome adversity associated with disclosure and HIV-related stigma and to consistently take their medications [12, 52, 54–56, 61, 67, 69, 72] :

I am a Christian and a believer, I know that God exists but those medicines also were inspired by God. God is the one who gave inspiration to doctors to make those medicines for us.

– 59-year-old man on ART, from the Democratic Republic of Congo [12, p. 4]

#### Theme 5: health systems

A theme common to several studies was that different aspects of the health system could help to moderate the impacts of HIV-related stigma on ART adherence. Specifically, compassionate human capital elements could establish a supportive clinical environment for patients, while certain clinical programs could be designed to address care for the entire family. As noted by one HIV-positive participant in Connecticut,

[The nurses] take care of me, I love the people, they go to your home, like they're my friends. Every time they say, how are you doing? Do you need anything? [75, p. 117].

Doctors and nurses engaged in patient-centred care could help to establish bonds of trust and empower patients to overcome the stigma associated with taking medications [50, 55, 60, 62, 63, 67–70, 72–75, 80]. Some participants described how medication regimens optimized for tolerability, with the fewest side effects and lowest pill burden, allowed them to minimize the possibility that others in the community might recognize their HIV status; this, in turn, decreased stigma and increased participants’ chances of successfully adhering to treatment [55, 58, 60, 73, 74, 80]. Lastly, family-driven treatment programs designed to bring all HIV-positive members of the family into care were thought of as cultivating greater social support, reducing stigma and improving ART adherence [54, 57].

### Synthesis of quantitative studies

Data from the quantitative studies were consistent with these lines of inquiry. Our systematic search protocol identified 34 cross-sectional and seven longitudinal studies conducted between 1997 and 2009 that examined the association between either stigma or disclosure and ART adherence (Table 2). These studies included data from 25,387 participants living in 18 different countries, with the largest proportion of studies (15/41 (37%)) based on data collected in the United States. The median number of participants was 300 (IQR, 201–439; range, 65–5760). Twenty-three studies (56%) measured HIV-related stigma, while 21 studies (51%) measured disclosure of seropositivity and three studies (7%) included a measure of both. Most of the studies examining the effect of HIV-related stigma (18/23 (78%)) on ART adherence employed a scale for which some evidence of reliability and/or validity had previously been obtained. In five studies, a multifactor scale was used (28%), while in others specific aspects of HIV-related stigma were measured, including enacted stigma (2/18 (11%)), disclosure concerns (3/18 (16%)), perceived stigma (3/18 (16%)) and internalized stigma (11/18 (61%)) (total percentage exceeds 100% as some studies administered more than one scale). Of the 18 studies that used a formal scale for measuring stigma, only three studies (17%) were conducted in a sub-Saharan African setting, and each of these used a newly developed stigma scale. The most widely used scale, administered in six studies, was the four-factor HIV Stigma Scale developed by Berger *et al*. [81]. To measure ART adherence, most studies used self-report (30/41 (73%)). Of these, slightly more than half (16/30 (53%)) employed a scale with previously demonstrated evidence of reliability or validity; the AIDS Clinical Trials Group measure developed by Chesney *et al*. [94] was the most frequently used among these (10/16 (63%)).

**Table 2:** Studies reporting a quantitative measure of association between stigma or disclosure and ART adherence (*N*=41)

Citation	Study design and population	Study period	Primary stigma or disclosure measure	Primary adherence measure	Findings
Birbeck *et al*. [82]	Cross-sectional study of 255 outpatients from 3 clinics in rural Zambia	2005–06	Disclosure of HIV seropositivity to spouse, family, friend, or no one	“Good adherence” was defined as (a) attendance at all ART clinic visits, (b) no lapse in drug collection, and (c) no clinic documentation indicating adherence problems	Of those who had not disclosed to anyone, only 17% had good adherence, whereas 50–66% of those who had disclosed to a spouse, family member or friend had good adherence (*p*=0.047)
Adeyemi *et al*. [83]	Cross-sectional study of 320 outpatients on ART for at least 12 months, recruited in 2 cities in Nigeria	2009	Unclear measure (“stigma and discrimination”)	Greater than one week delay in ART refill, as determined by comparison of date of scheduled appointment and date of actual refill	“Stigma and discrimination” was associated with increased odds of delayed ART refill (AOR=1.4; 95% CI=1.1–1.7), after adjusting for distance to clinic and occupation
Boyer *et al*. [84]	Cross-sectional study of 2381 inpatients in 27 national, provincial and district hospitals throughout Cameroon	2006–07	Personal experience of HIV-related stigma from partner or close family members	Self-reported ART adherence based on a 14-item scale related to dose-taking and dosing schedule [85], with “non-adherent” persons defined as those who had taken<100% of prescribed doses in the past four weeks but did not report any treatment interruptions lasting>2 consecutive days	Experience of discriminatory behaviours was associated with increased odds of non-adherence (AOR=1.74, 95% CI=1.14–2.65), after adjusting for household income, binge drinking, food insecurity, social support and healthcare supply-related factors
Cardarelli *et al*. [86]	Cross-sectional study of 103 outpatients at a preventive medicine clinic for low-income persons in Texas	2008^a^	40-item HIV stigma scale [81]	Non-adherence was defined as a positive screen on the simplified medication adherence questionnaire, a modified version of the Morisky scale, which contains 6 items related to forgetfulness or carelessness about ART dose taking behavior [87, 88]	The stigma score did not have a statistically significant association with non-adherence (AOR=1.01; 95% CI=0.98–1.03), after adjusting for race, education, racial discrimination, social support, perceived stress or sense of control
Carlucci *et al*. [89]	Cross-sectional study of 424 outpatients at a mission hospital in rural Zambia	2006	Single-item question about perceived stigma	Pill count adherence measured over a median of 84 days (interquartile range, 56–98 days), with optimal adherence defined as≥95% doses taken	Perceived stigma did not have a statistically significant association with adherence (AOR=1.1; 95% CI=0.55-2.1), after adjusting for travel time and transportation cost
Charurat *et al*. [90]	Cross-sectional study of 5760 persons initiating ART at five university teaching hospitals in urban Nigeria	2005–06	HIV disclosure to spouse or family members	Pharmacy refill adherence rate (days of medication dispensed divided by days between visits), with poor refill adherence defined as<95% adherence	Disclosure was associated with decreased odds of low adherence (AOR=0.85; 95% CI=0.75–0.97), after adjusting for education, employment, distance to clinic
					and time on ART. There was no univariable association with loss to follow up (OR=0.96; 95% CI=0.82–1.12)
Colbert [91]	Cross-sectional analysis of baseline data on 335 persons participating in a 5-year randomized clinical trial conducted in clinics and HIV service organizations in western Pennsylvania and northeast Ohio	2003–07	40-item HIV stigma scale [81]	30-day adherence as measured with electronic event monitoring, with poor adherence defined as<85% adherence	Neither personalized stigma (AOR=0.98; 95% CI=0.95-1.02) nor negative self-image (AOR=1.00; 95% CI=0.94–1.06) had a statistically significant association with poor adherence, after adjusting for mental health, self-efficacy and health literacy
Diiorio *et al*. [92]	Cross sectional study of 236 outpatients (32% women) from an HIV clinic in Atlanta	2001–03	Four items related to internalized stigma from the Perceived Stigma of HIV and AIDS Scale [93]	Five items related to logistical adherence barriers from the ACTG Adherence Instrument [94]	In a structural equation model, stigma had an indirect negative association with adherence: stigma was found to erode self-efficacy, which in turn was directly associated with adherence
Dlamini *et al*. [95]	Longitudinal study of 698 persons (72.3% on ART for more than 1 year) enrolled in a larger cohort in Lesotho, Malawi, South Africa, Swaziland and Tanzania	2006–07	33-item HIV and AIDS Stigma Instrument-PLWA [96]	ACTG Adherence Instrument [94]	Persons who did not report any missing doses experienced a steeper decline in mean stigma over time, after adjusting for education, employment, food insecurity, social support and years since diagnosis
Do *et al*. [97]	Cross-sectional study of 300 outpatients from the largest ART clinic in Botswana	2005	Disclosure of seropositivity to a partner	Adherence defined as no missed doses with four-day and one-month recall, and no missed refill visits with 90-day recall	Non-disclosure was associated with an increased odds of non-adherence (*p*<0.02; AOR not shown), after adjusting for education, employment, travel time, duration of ART, depression, alcohol use and household size
Franke *et al*. [98]	2-year longitudinal study of 134 adults initiating ART in urban Peru	2005–09	40-item HIV stigma scale [81]	30-day self-report, with “suboptimal” adherence defined as<95% [94]	On univariable analysis, perceived HIV stigma was not associated with suboptimal adherence (OR=1.03, 95% CI 0.94–1.12) and was not included in the final multivariable model
Goldman *et al*. [99]	Longitudinal study of 913 treatment-naïve adults initiating ART in urban Zambia	2006–07	Disclosure of HIV status to partner or spouse	Medication possession ratio based on cumulative days late for pharmacy refill visits, with≥95% defined as optimal adherence	Disclosure did not have a statistically significant association with optimal adherence (estimates not reported)
Kalichman *et al*. [100]	Cross-sectional study of 81 adults recruited from HIV clinical and community support services in Atlanta	2005^a^	4-item self-efficacy for disclosure decisions scale	6-item standard medication adherence self-efficacy scale [101]	Self-efficacy for disclosure had a statistically significant correlation with self-efficacy for engaging in care (*r*=0.24, *p*<0.05) but not with self-efficacy for medication adherence (*r*=0.19, p>0.05)
Kalichman *et al*. [102]	Cross-sectional study of 145 adults recruited from HIV clinical and community support services in Atlanta	2008^a^	6-item Internalized AIDS-Related Stigma Scale [103]	Monthly unannounced pill count conducted by telephone, averaged over four months, with adherence defined as ≥85% of doses taken	Internalized stigma had no statistically significant association with adherence (AOR=0.99, 95% CI 0.87–1.13)
Li *et al*. [104]	Cross-sectional study of 386 adults (23.9% of whom were treatment-naïve), recruited from four district hospitals throughout Thailand	2007	8-item scale assessing serostatus disclosure to various social ties [105] and 9-item internalized stigma scale [106, 107]	30-day self-reported adherence, with good adherence defined as no missed doses	Good adherence had a statistically significant association with disclosure (AOR=1.70; 95% CI=1.07–2.70) but not internalized stigma (AOR=0.83; 95% CI=0.51–1.36), after adjusting for education, employment, instrumental social support, depression symptom severity, family functioning and years since diagnosis
Li *et al*. [108]	Cross-sectional study of 202 outpatients enrolled in the Chinese national free ART program, selected from six HIV treatment sites in Hunan Province, China	2009	34-item, five-factor HIV-related stigma scale [109]	Seven-day self-reported ART adherence as measured on a 5-point Likert scale [110]	Stigma was associated with a reduced odds of good adherence (AOR=0.96; 95% CI=0.93–0.98), after adjusting for education, family income, years since diagnosis and recent drug use
Lucero *et al*. [111]	Cross-sectional study of 65 persons aged >50 years recruited from two hospitals in New York City	2001^a^	Disclosure of HIV seropositivity to family and friends	Self-report, rated on a 4-point Likert-type scale, with good adherence defined as “taking medication all of the time”	Disclosure was associated with better adherence (estimates not shown)
Martinez *et al*. [112]	Longitudinal study of 178 girls and women aged 15-24 years recruited from 5 cities throughout the U.S.	2003–05	The disclosure concerns and negative self-image subscales of the HIV stigma scale [81]	12-item scale to measure self-reported dosing and scheduling adherence with a two-day recall	Baseline stigma did not have a statistically significant association with complete adherence at 12-month follow-up (*b*=−0.012, *p*>0.50).
Mo and Mak [113]	Cross-sectional study of 102 adults recruited from an outpatient clinic in Hong Kong	2009^a^	22-item self-stigma scale [114]	ACTG Adherence Instrument [94], with participants classified as “adherers,” “unintentional non-adherers,” or “intentional non-adherers”	Intentional non-adherers had greater self-stigma (4.11, SD 0.74) than adherers (3.78, SD 0.96) and unintentional non-adherers (3.22, SD 0.92) F[1,100]=7.58, *p*<0.001)
Molassiotis *et al*. [115]	Cross sectional study of 136 adults recruited from an outpatient clinic in Hong Kong	2002^a^	HIV disclosure to others, including spouses or partners	ACTG Adherence Instrument [94], with good adherence defined as≥95% adherence	Disclosure did not have a statistically significant association with adherence (estimates not shown)
Muyingo *et al*. [116]	Secondary analysis of data from a randomized trial of 2957 treatment-naïve adults initiating ART at two treatment centres in Uganda and one in Zimbabwe	2003–04	Disclosure of HIV serostatus	Drug possession ratio, with complete adherence defined as 100% adherence	Disclosure did not have a statistically significant association with complete adherence (estimates not shown), after adjusting for education and duration of current partnership
Nachega *et al*. [117]	Cross-sectional study of 66 outpatients at an HIV clinic in South Africa	2002	Fear of stigma from partner	ACTG Adherence Instrument [94]	On univariable analysis, fear of stigma from partner was associated with reduced odds of >95% adherence (OR=0.13; 95% CI=0.02–0.70)
Olowookere *et al*. [118]	Cross sectional study of 318 adults on ART for at least three months, recruited from a university hospital HIV clinic in Nigeria	2007	Disclosure of HIV serostatus	Seven-day self-reported adherence, with non-adherence defined as<95% doses taken	Non-disclosure was associated with increased odds of non-adherence (AOR=1.7; 95% CI=1.0–2.8), after adjusting for transportation costs
Peltzer *et al*. [119]	Cross-sectional study of 735 adults newly initiating ART at one of 3 public hospitals in KwaZulu-Natal, South Africa	2007–08	7-item version of the AIDS-Related Stigma Scale [120], modified to reflect internalized stigma; 7-item AIDS-related discrimination scale	ACTG Adherence Instrument [94] and 30-day visual analogue scale [121], with partial or full adherence defined as ≥95% adherence	Partial or full VAS adherence was associated with AIDS-related discrimination (AOR=0.60; 95% CI=0.46–0.78) but not internalized stigma (OR=1.11; 95% CI=0.97–1.27), after adjusting for alcohol use and social support; use of the ACTG Adherence Instrument yielded similar results
Penniman [122]	Secondary analysis of baseline data on 259 women enrolled in a larger cohort study in Los Angeles	2005–06	Disclosure of HIV serostatus to child	3-item self-reported dose-taking and timing adherence with two-day recall	Non-disclosure was associated with reduced odds of adherence (AOR=0.46; 95% CI=0.24–0.88), after adjusting for stress, family functioning and depression symptom severity
Peretti-Watel *et al*. [123]	Cross-sectional study of 2932 adults recruited from 102 hospitals in France	2003	Disclosure of HIV serostatus to friends and family; HIV-related discrimination by friends or family	Self-reported measure based on dose and timing adherence with one-week recall, with “high adherence” defined as no doses missed or mistimed	Poor adherence was associated with HIV-related discrimination (AOR=1.68; 95% CI=1.00–2.82) but not selective disclosure to significant others (AOR=0.73; 95% CI=0.28–1.94), after adjustment for alcohol and drug use
Rao *et al*. [124]	Cross-sectional study of 720 outpatients from a university HIV clinic in Seattle	2009	Summated rating scale of 4 items related to internalized and enacted stigma, from the 24-item Stigma Scale for Chronic Illness [125]	3 items from the ACTG Adherence Instrument [94], a one-item rating response measure [126] and a 30-day VAS [121]	In a structural equation model, stigma was associated with reduced adherence (*b*=–0.21, *p*<0.01); the authors concluded that the effect was mediated by depression symptom severity
Rintamaki *et al*. [127]	Cross-sectional study of 204 outpatients at two urban academic medical centre clinics in Illinois and Louisiana	2001	Summated rating scale of 3 items from the Patient Medication Adherence Questionnaire (PMAQ) [128, 129] related to internalized stigma and disclosure concerns	Non-adherence defined as any missed doses in the prior four days, assessed using the PMAQ	High stigma was associated with greater odds of non-adherence (AOR=3.3; 95% CI=1.4–8.1), after adjusting for race & education
Rotheram-Borus *et al*. [130]	Secondary analysis of baseline data from a randomized controlled trial of 409 adults recruited from 4 district hospitals in northern Thailand	2009^a^	7-item summative rating scale assessing extent of HIV serostatus disclosure to social network ties	Self-reported lifetime adherence, with good adherence defined as never having missed a dose	Disclosure had a statistically significant association with adherence (*b*=0.11, *p*<0.05); the authors concluded that disclosure operates primarily through its effect on family functioning
Rougemont *et al*. [131]	Longitudinal study of 312 treatment-naïve adults initiating ART in Yaoundé, Cameroun	2006–07	Disclosure of HIV serostatus to family	Pharmacy refill, with “non-adherers” defined as “renewal of prescriptions of later than two weeks”	Non-disclosure did not have a statistically significant association with non-adherence (AOR=0.98; 95% CI=0.81–1.18), after adjustment for income, education and distance to clinic
Sayles *et al*. [132]	Cross-sectional study of 202 adults recruited from 5 community organizations and 2 HIV clinic sites in Los Angeles	2007	28-item internalized stigma scale [133]	Seven-day self-reported ART adherence as measured on a 5-point Likert scale [110], with suboptimal adherence as defined as any response other than “all of the time”	A high level of internalized stigma was not associated with suboptimal adherence (AOR=2.09; 95% CI=0.81–5.39), after adjusting for mental health, race, education, income, insurance and years since diagnosis
Spire *et al*. [134]	Longitudinal study of 445 treatment-naïve adults initiating ART, recruited from 47 hospitals across France	1997	Disclosure of HIV serostatus to a family member	Self-reported adherence over prior four days, with “adherent” defined as 100% adherence	71% of participants who had disclosed to a family member at baseline were classified as adherent four months later, compared to 76% of those who had not disclosed (*p*=0.26)
Stirratt *et al*. [135]	Cross-sectional study of 215 adults recruited from 2 outpatient HIV clinics in New York City	2000–04	Disclosure of HIV serostatus to up to 15 family members and 15 personal contacts [136]	14-day ART adherence as measured by electronic event monitoring	Percentage of informed family members had a statistically significant association with ART adherence (*b*=0.21, *p*<0.05)
					after adjusting for self-efficacy, motivation and outcome expectancies
Sumari-de Boer *et al*. [137]	Cross-sectional study of 201 outpatients at an academic medical centre HIV clinic in Amsterdam, the Netherlands	2008–09	Personalized stigma and disclosure concerns sub-scales of the HIV stigma scale [81]	30-day pharmacy refill adherence, with non-adherence defined as<100% adherence	Non-adherence had a statistically significant association with disclosure concerns (AOR=1.1; 95% CI=1.01–1.2) but not personalized stigma (AOR not reported), after adjusting for years since diagnosis, quality of life and depression symptom severity
Van Dyk [138]	Cross-sectional study of 439 adults recruited from public health HIV clinics and hospitals in Pretoria, South Africa	2008	Disclosure of HIV serostatus to partner	30-day self-reported adherence as elicited through a visual assessment scale [121], with optimum adherence defined as >90% adherence	41% of participants who had disclosed to partners reported optimum adherence, compared to 21% of participants who had not disclosed (*p*=0.006)
Vanable *et al*. [139]	Cross sectional study of 221 outpatients in central New York state	2001	Five-item frequency of stigma-related experiences scale	Summary self-reported adherence measure averaged across 4 items based on a seven-day recall period	Stigma-related experiences had a negative association with self-reported adherence (*b*=−0.20, *p*<0.01), after adjusting for income, employment status and time since diagnosis
Waite *et al*. [140]	Cross-sectional study of 204 outpatients at two urban academic medical centre clinics in Illinois and Louisiana	2001	Summated rating scale of 3 items from the Patient Medication Adherence Questionnaire (PMAQ) [128, 129] related to internalized stigma and disclosure concerns	Non-adherence defined as any missed doses in the prior four days, assessed using a modified version of the PMAQ	A high level of stigma was associated with increased odds of non-adherence (AOR=3.1; 95% CI=1.3–7.7), after adjusting for insurance coverage, employment, mental disorder and history of alcohol or drug treatment
Wang *et al*. [141]	Cross-sectional study of 308 adults recruited from seven treatment sites in China	2006	Disclosure of HIV serostatus	Seven-day self-reported adherence, with good adherence defined as>90% of doses taken	Disclosure did not have a statistically significant association with adherence (estimates not shown)
Watt [142]	Cross sectional study of 340 persons in Tanzania	2007^a^	10-item perceived stigma scale [143], and number of social network ties to whom the participant had disclosed his or her seropositivity	Self-reported missed doses in the prior four days [94], and 30-day self-reported adherence using a modified visual analogue scale [121], with optimal adherence defined as≥95% adherence on both instruments	On univariable analysis, neither stigma nor disclosure had statistically significant associations with optimal adherence (estimates not shown)
Weiser *et al*. [144]	Cross-sectional study of 109 persons recruited from three private clinics in Botswana	2000	Disclosure of HIV serostatus	12-month self-reported adherence [94], with good adherence defined as≥95% of doses taken	On univariable analysis, disclosure did not have a statistically significant association with good adherence (OR=3.55; 95% CI=0.91–13.92)
Wolitski *et al*. [145]	Cross-sectional study of 637 homeless or unstably housed persons in three U.S. cities	2004	Modified 6-item internalized and 6-item perceived HIV stigma scales [81]	Self-reported missed doses in the prior two days and seven days	Perceived stigma, but not internalized stigma, was associated with increased odds of missed doses in the past two days (AOR=1.40; 95% CI=1.00–1.95) and past seven days (AOR=1.41; 95% CI=1.05–1.89), after adjusting for housing status, education, and years since HIV diagnosis

a Refers to date of publication, as dates of data collection were not clearly described.

Among the 41 studies, 25 (61%) reported a positive finding (i.e., showing that stigma was associated with reduced ART adherence or that disclosure was associated with improved adherence) while 16 (39%) reported a null finding. No studies reported that better ART adherence was paradoxically associated with greater intensity of stigma or less disclosure. A roughly equal proportion of studies conducted outside of the United States reported a positive finding compared to US-based studies (16/26 (62%) vs. 9/15 (60%); Pearson's *χ* ^2^=0.01, *p*=0.92).

When the studies were disaggregated by study design, most of the cross-sectional studies (24/34 (71%)) reported a positive finding, while most of the longitudinal studies (6/7 (86%)) reported a null finding (Pearson's *χ* ^2^=7.7; *p*=0.005). When disaggregated by exposure, these differences were slightly attenuated. Among studies examining the impact of a stigma variable on adherence, 15/20 (75%) cross-sectional studies vs. 1/3 (33%) longitudinal studies reported a positive finding (Pearson's *χ* ^2^=2.14; *p*=0.14). Among studies examining the impact of disclosure on adherence, 11/17 (65%) cross-sectional studies vs. 0/4 (0%) longitudinal studies reported a positive finding (Pearson's *χ* ^2^=5.4; *p*=0.02).

In three cross-sectional studies, the authors fit structural equation models to investigate the relationships between study variables. Diiorio *et al*. [92] concluded that the association between stigma and ART adherence was mediated by self-efficacy: perceived stigma eroded one's confidence about adhering to a treatment regimen, which in turn undermined treatment adherence. Rao *et al*. [124] did not measure self-efficacy but concluded that internalized stigma worsened symptoms of depression, like fatigue and concentration difficulties, which in turn compromised one's ability to adhere to a complex treatment regimen. In the study by Rotheram-Borus *et al*. [130], disclosure had a statistically significant association with ART adherence; the authors concluded that the effect was mediated principally by improvements in family function.

## Conceptual model

**Figure 3:**
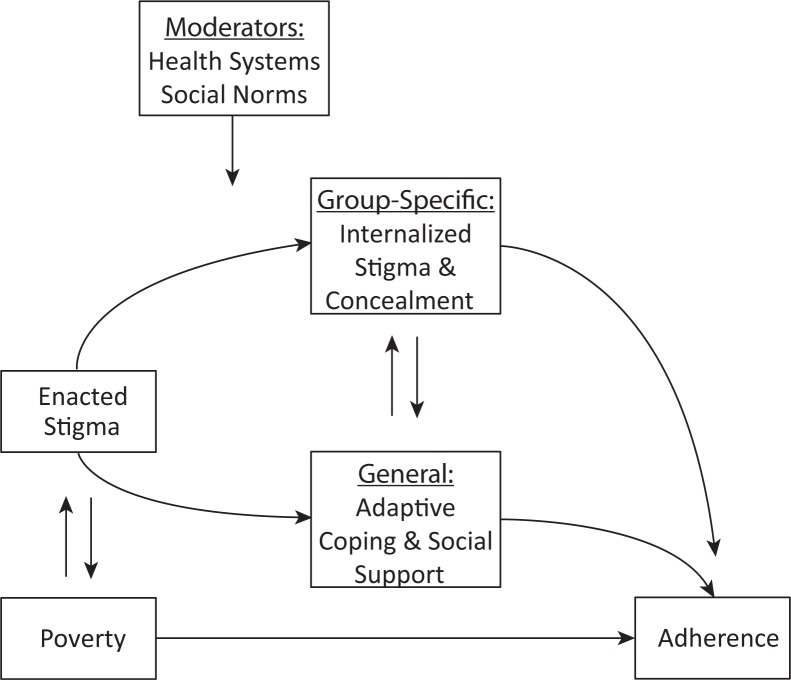
Conceptual model. This figure summarizes the findings of our meta-synthesis of 34 qualitative studies and analysis of 41 quantitative studies. The stigma of HIV was found to compromise ART adherence through general as well as group-specific psychological processes. Adaptive coping and social support were critical determinants of participants’ ability to overcome structural and economic barriers associated with poverty to successfully adhere to ART.

To integrate our core findings from the qualitative and quantitative studies, we propose a conceptual model described in Figure 3, citing areas of congruence between our empirically derived themes and theoretical frameworks previously published by others. In our model, structural and economic barriers associated with poverty undermine ART adherence. Enacted stigma undermines ART adherence through psychological processes specific to HIV-positive persons as well as through general psychological processes that are common to HIV-positive and HIV-negative persons alike. Stigma and poverty have mutually reinforcing relationships with each other, particularly in resource-limited settings [146]: stigma and social isolation have adverse economic impacts and, conversely, poverty worsens stigma by highlighting the economic aspects of HIV's perceived association with premature morbidity and mortality.

Internalized stigma may result when HIV-positive persons accept as valid the stigmatizing beliefs of the majority group. Because HIV infection is a potentially concealable stigma, HIV-positive persons may attempt to delay disclosure until disease progression renders further concealment impossible [147]. As elaborated in the stress process model [148, 149] and as described by the participants in the studies summarized in this review, HIV-positive persons draw on adaptive coping and social support to minimize the harmful effects of life stressors.

Adaptive coping and social support partially moderate the harmful effects of poverty on adherence and are represented in the diagram as effect modifiers: in the presence of adaptive coping or strong social support networks, the negative impacts of poverty on adherence are reduced. In this regard our synthesis is consistent with the social support model described by Ware *et al*. [150], who found that HIV-positive persons in Nigeria, Tanzania and Uganda relied heavily on social support to overcome structural and economic barriers to care. The authors concluded that the stigma of HIV was feared specifically because it weakened relationships that proved to be critical for everyday survival. In addition, as supported by both the qualitative and the quantitative studies summarized in this review, these general and group-specific psychological processes can directly benefit or undermine ART adherence. For example, in the setting of enacted stigma, many HIV-positive participants adopted strategies of concealment, which led directly to treatment interruptions.

The qualitative studies we identified also suggested a number of extensions to the model, namely that certain factors can moderate the severity of enacted stigma and their ultimate impacts on ART adherence. One such factor is the health system, which can be configured to support patients and minimize the harmful influences of stigma on ART adherence. Although resistance to stigma has been described [151], in countries with fragile healthcare systems resistance to stigma can be weakened as HIV-positive persons struggle with the anxieties of uncertain and unstable access to treatment [80]. Another factor involves social norms, which were described by participants in the qualitative studies as potentially intensifying the harmful influences of stigma. HIV-positive persons who belonged to sexual minority groups or who had acquired HIV through socially unacceptable means, in particular, experienced greater stigma because their self-identities and behaviours were defined by the majority as being inconsistent with social norms.

## Discussion

In this systematic review of both qualitative and quantitative studies conducted among 26,715 HIV-positive persons living in 32 countries worldwide, we found that HIV-related stigma compromised ART adherence, primarily by undermining social support and adaptive coping. Our analysis is consistent with prior work demonstrating the importance of social ties in promoting adherence, particularly in resource-limited settings [33, 152], and reflects the centrality of social integration to the experience of HIV-positive persons engaged in treatment. These themes are all the more prominent in settings of extreme poverty where treatment barriers are highly prevalent [8, 14, 153] and where social ties may be essential for survival [72, 154, 155]. Our findings have implications for public health strategies now being explored in high-HIV prevalence regions, such as universal voluntary testing with immediate treatment [36]. The evidence search protocol was not designed to identify studies examining the influences of stigma on HIV testing [156, 157], pre-ART linkage to care [158, 159], ART refusal [160], or other treatment- and care-related behaviours along the entire continuum of engagement in care [35]. However, HIV-related stigma has been shown to adversely affect these treatment- and care-related behaviours in a wide range of settings [35, 161–166]. Optimization of the entire continuum of care is needed to maximize the public health impact of test-and-treat [34], thereby underscoring the importance of our findings.

Several limitations are important to consider when assessing this systematic review. First, it is well known that qualitative studies can be difficult to locate using conventional search strategies [167]. Although we adopted a purposefully broad search protocol that involved the full text review of 960 journal articles, unpublished dissertations and conference abstracts, we cannot exclude the possibility that we may have missed some relevant studies. Second, and related to the previous, we only identified one (qualitative) study from the UNAIDS Eastern Europe and Central Asia region. The HIV epidemic follows a different pattern in these countries, with concentrated epidemics most notably driven by injection drug use but also by prison overcrowding and unprotected sexual intercourse among men who have sex with men and sex workers [168–170]. For people belonging to these already marginalized subgroups, the stigma of their HIV serostatus is layered upon these pre-existing inequalities, thereby displacing them further downward in the status hierarchy. If we had been able to identify more studies from this region, it is possible that different themes could have been identified in the qualitative synthesis or that an even stronger association between stigma and ART adherence would have been described. Third, heterogeneity in the types of exposures and outcomes used in the quantitative studies precluded a formal meta-analysis. The vote counting-styled procedures we employed to synthesize their findings could not generate effect size estimates, are characterized by low statistical power [171] and cannot assess the magnitude of the purported relationship. As the field converges on the use of standardized and validated measures of stigma, disclosure and adherence, we expect that the methods of meta-analysis can be increasingly applied. Fourth, a greater proportion of longitudinal studies reported a null association between ART adherence and either stigma or disclosure. The difference appeared to be driven by studies examining the impact of disclosure on adherence. The single longitudinal study that documented a positive finding employed validated instruments to measure both stigma and self-reported ART adherence, but in general the relatively small number of longitudinal studies limited our ability to draw strong conclusions. Fifth, the majority of studies included in this review were assessed to be at risk of bias. A key reporting deficiency in the qualitative studies was lack of detail on the method of analysis. The majority of quantitative studies did not use validated exposure and outcome measures. Although these factors could exert unpredictable biases, we acknowledge they could have biased the qualitative and quantitative findings towards the null, with attendant effects on our conceptual model.

These caveats aside, the conceptual model that emerged from our synthesis of the literature has several important implications for programming and policy. At the individual level, interventions focused on enhancing social support by activating [172] or strengthening existing ties [173, 174], or facilitating either of these through the encouragement of serostatus disclosure [175–177], may be expected to improve ART adherence. These behaviours may in turn yield health and mental health dividends. Although our meta-synthesis highlighted positive self-identity as an important factor related to greater adherence, more research is needed to understand the conditions under which HIV-related outcomes are better than expected despite the experiences of HIV- and stigma-related adversity (which can be thought of as being related to the concept of resilience [178–180]). It should be acknowledged here that social ties are not uniformly beneficial. This was observed in our data showing that all relationships were not necessarily described as supportive and that some study participants’ experiences suggested positive benefits from concealment. There have been few intervention studies where disclosure was emphasized as a primary outcome [181], but the outcomes of HIV serostatus disclosure are not unambiguously positive. Due to HIV-related stigma, significant others may react in negative ways after learning about a loved one's seropositivity [182–184]. In order to avoid these undesirable outcomes, interventions targeting disclosure behaviours should be sensitive to these potential negative consequences.

At the structural level, our model suggests that structural interventions (which target the context in which people live, including social ties, resources and institutions [185]) to enhance the capacity of health systems for providing quality care may help to minimize the adverse effects of HIV-related stigma on ART adherence. Structural interventions that strengthen the livelihoods of HIV-positive persons may also be a promising avenue for subverting HIV-related stigma, particularly in resource-limited settings where contributing to local solidarity networks is a core social function [186] and where the economic impacts of HIV and AIDS have exacerbated both the instrumental and symbolic aspects of stigma attached to HIV [187]. Castro and Farmer [188] advanced the argument that “structural violence determines, in large part, who suffers from AIDS-related stigma and discrimination” (p. 55). Although some observers have speculated that economic strengthening or livelihood interventions may play a role in reducing HIV-related stigma [146], to our knowledge these hypotheses have not been formally tested [189, 190]. Related work suggests that these may spark a “virtuous” cycle: as stigma-related barriers are levelled and as HIV testing, treatment and other care-related behaviours become more widespread, the stigma of HIV and AIDS can be reduced [188, 191–195].

Notably, our conceptual model also suggests several promising points of intervention to improve ART adherence that *have not* consistently yielded benefits when tested for their impacts on ART adherence. For example, several studies described how effective treatment of depression could potentially improve treatment adherence, consistent with the positive prevention model elaborated by Sikkema *et al*. [196]. However, depression intervention studies have yielded mixed findings to date with regards to HIV treatment adherence outcomes [197–199]. Likewise social support interventions should also be expected to improve adherence, but these have also proved inconclusive [200–203]. The lack of consistent findings may potentially be explained by the fact that interventions targeting intrapersonal or interpersonal processes fail to address the larger social forces undermining adherence to HIV treatment. We emphasize here that the concepts embedded in our conceptual model span multiple levels of analysis [204, 205], ranging from intrapersonal processes (self-identity, coping), to interpersonal processes (social support, concealment), to structural factors (health systems, poverty, stigma). We therefore expect that interventions spanning multiple levels would yield the greatest impacts on reducing stigma [206], but these approaches have been rarely employed.

## Conclusions

In this review of both qualitative and quantitative studies, we found that HIV-related stigma compromises ART adherence through general as well as group-specific psychological processes. Adaptive coping and social support were critical determinants of participants’ ability to overcome structural and economic barriers associated with poverty to successfully adhere to ART. Our conceptual model, which integrates the results of both quantitative and qualitative studies, suggests that the effects of stigma operate at multiple levels (intrapersonal, interpersonal and structural). Interventions to reduce stigma should target these multiple levels of influence in order to have maximum effectiveness on improving ART adherence.
